# Genetic factors influencing the risk of multiple myeloma bone disease

**DOI:** 10.1038/leu.2015.342

**Published:** 2016-01-12

**Authors:** D C Johnson, N Weinhold, J Mitchell, B Chen, O W Stephens, A Försti, J Nickel, M Kaiser, W A Gregory, D Cairns, G H Jackson, P Hoffmann, M M Noethen, J Hillengass, U Bertsch, B Barlogie, F E Davis, K Hemminki, H Goldschmidt, R S Houlston, G J Morgan

**Affiliations:** 1 Division of Molecular Pathology, The Institute of Cancer Research, London, UK; 2 Myeloma Institute, University of Arkansas for Medical Sciences, Little Rock, AR, USA; 3 Department of Internal Medicine V, University of Heidelberg, Heidelberg, Germany; 4 Division of Genetics and Epidemiology, The Institute of Cancer Research, London, UK; 5 German Cancer Research Center, Heidelberg, Germany; 6 Center for Primary Health Care Research, Lund University, Malmö, Sweden; 7 Leeds Institute of Molecular Medicine, Section of Clinical Trials Research, University of Leeds, Leeds, UK; 8 Department of Haematology, Newcastle University, Newcastle-Upon-Tyne, UK; 9 Institute of Human Genetics, University of Bonn, Bonn, Germany; 10 Division of Medical Genetics, Department of Biomedicine, University of Basel, Basel, Switzerland; 11 Department of Genomics, Life & Brain Center, University of Bonn, Bonn, Germany; 12 National Center of Tumor Diseases, Heidelberg, Germany

## Abstract

A major complication of multiple myeloma (MM) is the development of osteolytic lesions, fractures and bone pain. To identify genetic variants influencing the development of MM bone disease (MBD), we analyzed MM patients of European ancestry (totaling 3774), which had been radiologically surveyed for MBD. Each patient had been genotyped for ~6 00 000 single-nucleotide polymorphisms with genotypes for six million common variants imputed using 1000 Genomes Project and UK10K as reference. We identified a locus at 8q24.12 for MBD (rs4407910, *OPG*/*TNFRSF11B*, odds ratio=1.38, *P*=4.09 × 10^–9^) and a promising association at 19q13.43 (rs74676832, odds ratio=1.97, *P*=9.33 × 10^–7^). Our findings demonstrate that germline variation influences MBD and highlights the importance of RANK/RANKL/OPG pathway in MBD development. These findings will contribute to the development of future strategies for prevention of MBD in the early precancerous phases of MM.

## Introduction

Multiple myeloma (MM) is a B-cell malignancy characterized by the expansion of clonal plasma cells in the bone marrow.^1, 2^ The disease is typified by varying numbers of osteolytic lesions that are the result of reduced osteoblastic and increased osteoclastic activity.^3, 4^ The presence of such bone lesions is a major criterion used to distinguish MM that requires treatment from precursor entities such as monoclonal gammopathy of undetermined significance and smoldering myeloma, which can be managed expectantly,^5^ as such, it is important to understand factors impacting on the development of MM bone disease (MBD). Although most patients develop osteoblastic lesions, a subset is unaffected by MBD, the reasons for which are not understood. Understanding the genetic mechanisms that are responsible for these differences in MBD is a pressing clinical issue, which has important implications for the development of novel treatments and in determining which patients might benefit from alternate bone therapies. In this respect, it has been shown in the setting of osteoporosis that heritable factors account for 50–85% of the variation in bone mineral density (BMD).^6, 7^ As the interaction of bone-forming cells with MM cells is central to the development of an osteolytic lesion and MM clonal growth, we hypothesized that germline variation could also contribute to the development of MBD. We have recently conducted genome-wide association studies (GWAS) of MM searching for susceptibility alleles.^8, 9, 10^ Linking these genetic data to the extent of MDB at baseline has allowed us to search for genetic variants influencing MBD risk.

## Methods

### Patients

We studied four independent cohorts of MM patients that had been the subject of previous GWAS^8, 9, 10, 11^ (Supplementary Figure 1): (i) My9 comprising 1205 MM cases from the UK Medical Research Council Myeloma-IX trial^12^ (ISRCTN68454111); (ii) My11 comprising 768 MM cases from the UK Medical Research Council Myeloma-IX trial^8^ (ISRCTN49407852); (iii) HdB, comprising 1182 MM patients recruited by the German-speaking Myeloma Multicentre Group (GMMG), coordinated by the University Clinic, Heidelberg^9^ (ISRCTN644552890, ISRCTN05745813); (iv) ArK, comprising 619 newly diagnosed MM patients treated at the UAMS Myeloma Institute, Little Rock, AR, USA^11^ (NCT00580372, NCT00081939, NCT00572169, NCT00734877). The clinical characteristics and demographics of the patients in each of the four patient cohorts are summarized in Table 1. There was a higher proportion of patients with WHO performance stage ⩾3 MM in My9 and My11, reflecting in part the increased age of patients in the non-intensive arms of these trials. The study was approved by the respective institutional ethical review boards: MREC 02/8/95 (My9); MREC 17/09/09 (My11); 229/2003, S-337/2009, AFmu-119/2010 (HdB) and all participants provided written informed consent.

### Radiological assessment of bone lesions

MBD was detected using axial survey in My9 and My11, axial skeletal survey (2001–2010) and whole body computed tomography (2011 onwards) in HdB and combined skeletal survey and skeletal computed tomography in ArK. The frequency of MBD was marginally higher in the HdB cohort (*P*=0.01). Owing to the differences in sensitivity of the radiological methods used to detect MBD, patients were classified as either affected (MBD) or unaffected (no MBD). Age was not significantly associated with MBD in any of the four cohorts (that is, *P*>0.05).

### Tumor karyotyping

Conventional cytogenetics of tumors was conducted using standard karotyping methodologies, and standard criteria for the definition of a clone were applied.^13^ Fluorescent *in situ* hybridization and ploidy classification of My9 and My11 samples were conducted using the methods described by Chiecchio *et al.*^14^ Fluorescent *in situ* hybridization analysis and ploidy classification of HdB samples were performed as previously described.^15^ The XL IGH Break Apart probe (MetaSystems, Altlussheim, Germany) was used to detect immunoglobulin H translocations in HdB samples.

### Genotyping and quality control

All cases were genotyped using Illumina Human OmniExpress arrays adhering to the manufacturer's protocols (Illumina, San Diego, CA, USA). Standard quality control was performed on all scans, excluding individuals with low call rate (<90%) and extremely high or low heterozygosity (*P<*1.0 × 10^−4^), as well as all individuals shown to be of non-European ancestry (using the HapMap version 2 CEU, JPT/CHB and YRI populations as a reference; Supplementary Figure 2). A summary of the number of genotyped single-nucleotide polymorphisms (SNPs) and the number of SNPs passing quality controls is shown in Supplementary Figure 1.

### Imputation

Genotypes for common variants across the genome were imputed using data from 1000 Genomes Project (phase 1 integrated release 3, March 2012) and UK10K as reference in conjunction with IMPUTE2 v2.1.1^16^ after pre-phasing with SHAPEIT software;^17^ poorly imputed SNPs defined by an information measure <0.90 were excluded. All genomic locations are given in National Center for Biotechnology Information Build 37/UCSC hg19 coordinates. All SNPs having a minor allele frequency<1% were excluded.

### Statistical analysis

We compared the relationship between genotype and presence or absence of MBD by logistic regression including covariates found in univariate analysis to influence MBD. We adjusted for the method of radiological assessment in each cohort and used eigenvalues in the analysis of the HdB series, to adjust for population substructure. *P*-values presented correspond to the significance of a test difference among all three of the genotype groups (common allele homozygote, heterozygote and rare allele homozygote). We confined our analysis to SNPs with a minor allele frequency >1% because of extreme value of the test statistics. Overall statistical significance tests for each SNP were performed by combining the results for each cohort by a fixed-effects meta-analysis. All statistical tests were two-sided. Inflation of the test statistics, *λ*, was estimated by dividing the 45th percentile of the test statistic by 0.357–the 45th percentile for a *χ*^2^ distribution on 1 degree of freedom. Between-study heterogeneity was quantified using the *I*^2^ statistic. Associations were regarded as statistically significant at a *P*-value ⩽5.0 × 10^–8^ (that is, genome-wide significance). All statistical analyses were performed using PLINK v1.07^18^ and R (v3.1.3) software.^19^

### Functional prediction

To explore the epigenetic profile of genomic location associated with MBD, we used ENCODE histone modification data and HaploReg and RegulomeDB^20, 21^ to examine whether any of the SNPs or their proxies (that is, *r*^2^>0.8 in the 1000 Genomes European reference panel) annotate transcription factor binding or enhancer elements. We assessed sequence conservation using Genomic Evolutionary Rate Profiling scores.^22^

## Results

### Relationship between genotype and bone lesions

After quality control measures were applied, genotype data on 55 31 610 SNPs was available for 3774 MM cases with MBD data. Quantile–Quantile plots of test statistics of the relationship between SNP genotype and MBD for each of the four cohorts is shown in Supplementary Figure 3; inflation factors *λ*=1.002–1.01, *λ*=1.0008 for the meta-analysis (Supplementary Figure 4).

**Figure 1:**
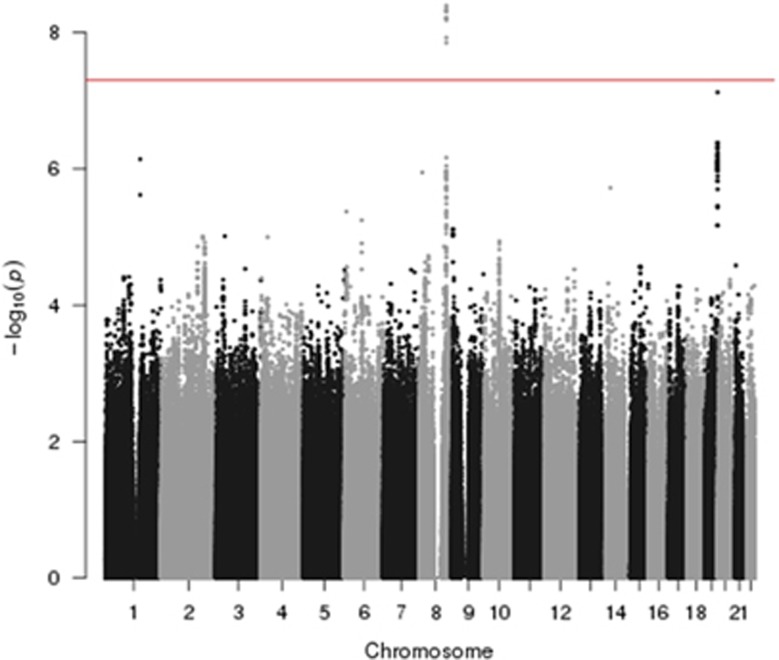
Association plot for combined analyses. The *P*-values of the association between each single-nucleotide polymorphism (SNP) and MBD. The *y* axis shows the −log_10_ *P*-values of each SNP analyzed, and the *x* axis shows their respective chromosome position. The red horizontal line corresponds to *P*=5.0 × 10^−^^8^. All statistical tests were two-sided.

**Figure 2:**
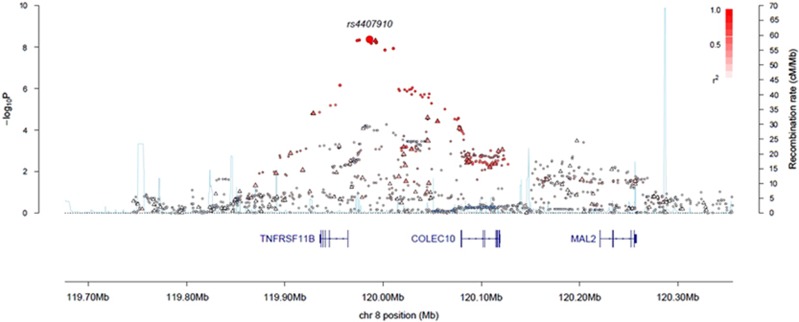
Regional plot of association and recombination rates for the 8q24.12 locus. Plots show association results of both genotyped (triangles) and imputed (circles) SNPs and recombination rates. −log_10_ *P*-values (*y* axes) of the SNPs are shown according to their chromosomal positions (*x* axes). rs4407910 shown as a large diamond. The color intensity of each symbol reflects the extent of LD with rs4407910 white (*r*^2^=0) through to dark red (*r*^2^=1.0). Genetic recombination rates, estimated using HapMap samples from Utah residents of western and northern European ancestry (CEU), are shown with a light blue line. Physical positions are based on NCBI build 37 of the human genome. Also shown are the relative positions of genes and transcripts mapping to the region of association.

**Figure 3:**
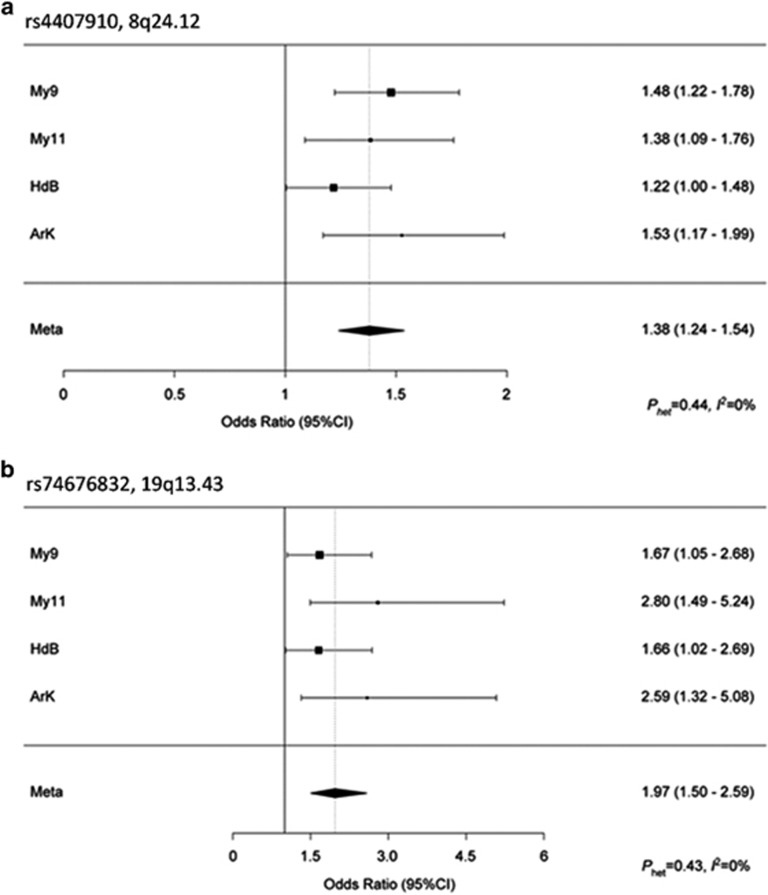
Forest plot of the ORs for the association between (**a**) rs4407910, (**b**) rs74676832 and MBD. Studies were weighted according to the inverse of the variance of the log of the OR calculated. Horizontal lines: 95% CI. Box: OR point estimate; box area is proportional to the weight of the study. Diamond (and broken line): overall summary estimate, with CI given by its width. Unbroken vertical line: null value (OR=1.0).

Nine SNPs showed an association with MBD and reached genome-wide significance (Figures 1 and 2, Supplementary Table 1). All nine SNPs were located in the same region at 8q24.12 and were in strong linkage disequilibrium (LD). The strongest association at 8q24.12 was provided by the common SNP rs4407910 (risk allele frequency=0.50, odds ratio=1.38, 95% confidence interval=1.24–1.54, *P*=4.02 × 10^−9^). The association was consistent across each of the four patient cohorts (Figure 3 and there was no significant between-study heterogeneity (*P*_het_=0.44, *I*^2^=0%). An increased prevalence of MBD has been observed in male patients and hyperdiploid MM.^23, 24^ Stratifying data by sex or ploidy did not provide evidence for a differential effect of rs4407910 genotypes on the risk of MBD (Supplementary Figures 5 and 6). Variation at 8q24.12 has been previously associated with BMD and osteoporosis, but not as strongly with risk of fracture.^25, 26, 27, 28^ Meta-analysis of My9 and My11 data showed no evidence that rs4407910 genotype influenced the risk of vertebral body fracture (*P*=0.28).

The MBD risk SNP rs4407910 localizes 19Kb 3' to the gene encoding *TNFRSF11B* (tumor necrosis factor receptor superfamily, member 11b; alias osteoprotegerin; *OPG*). The genomic region contains multiple enhancer markers from several tissue types including bone marrow cells (Supplementary Table 2 and Supplementary Figure 7). In lymphoblast and other tissues, rs4355801, which is in perfect LD with rs4407910 (*r*^2^=1.0, *D*'=1.0) is associated with *OPG* expression; the risk allele for MBD being associated with reduced *OPG* expression (Supplementary Table 3).^29, 30, 31, 32^

In addition to rs4407910, we detected a promising association for MBD marked by rs74676832 at 19q13.43 (odds ratio=1.97, 95% confidence interval=1.50–2.59, *P*=9.33 × 10^–7^, *P*_het_=0.43, *I*^2^=0%). rs74676832 is located within a 29 kb region of LD intergenic to *ZNF444* and *GALP* (Figures 1 and 3 and Supplementary Figure 8).

### Impact of alleles influencing BMD on MM bone disease

Variation at 8q24.12 marked by rs23062375 and rs11995824, which are intronic SNPs in *OPG* and in LD with rs4407910 (respective LD metrics *r*^2^ and *D'* – 0.78, 0.93 and 0.79, 1.0) have been demonstrated to influence BMD.^25, 26, 27, 31, 33^ To explore the possibility that other genetic variants influencing BMD also influence MBD, we investigated the association at 77 established risk loci for BMD with MBD^25, 26, 27, 28^ (Supplementary Table 4). Aside from the 8q24.12 SNPs, no other BMD locus showed an association with MBD after adjusting for multiple testing (that is, *P*>0.001). Moreover, there was no over-representation of association for MBD across the 77 SNPs (Supplementary Table 5).

## Discussion

Our findings support the hypothesis that an individual's risk of developing MBD is influenced by germline variation. Specifically, we identified a locus at 8q24.12 (rs4407910) associated with MBD. rs4407910 maps to a region of LD that only contains *OPG*, and when taken in conjunction with eQTL data it is likely that reduced *OPG* expression is the functional basis of the 8q24.12 association.^31^

Osteoclasts (OCs) are bone-resorptive cells, which are critical for the integrity of bone. OC-differentiation and activation is dependent on activation of nuclear factor-kB ligand (RANKL) signaling through the p38 MAPK pathway.^34, 35, 36^ OPG is a negative regulator of bone resorption acting as a decoy receptor for RANKL, decreasing bone resorption through inhibiting differentiation of OC precursors, activating mature OCs and stimulating OC apoptosis. Germline inactivating mutations in *OPG* are responsible for the autosomal dominant diseases: early-onset and familial Paget'disease, familial expansile osteolysis and expansile skeletal hyperphosphatasia, which are characterised by the development of expansile osteolytic bone lesions.^37, 38^ Myeloma cells express RANKL and treatment of mice models of MM with OPG has been demonstrated to prevent lytic bone lesions, maintaining cancellous bone volume and inhibiting OC formation.^12, 32^

Current clinical management of MBD involves reducing myeloma cell infiltration of the bone marrow using bisphosphonates to inhibit OC activity; however, there remains a need to develop more targeted treatments. Established MM therapies such as immunomodulatory drugs and proteasome inhibitors partially exert their activity through the OPG/RANK/RANKL system.^39, 40^ Hence, targeting the OPG/RANK/RANKL system through specific agents such as raloxifene may have therapeutic potential.^41, 42^ Raising OPG levels directly by infusion of recombinant OPG (Fc-OPG) suppresses bone resorption.^43^ Potential concerns over the generation of anti-Fc-OPG and binding to TRAIL has however shifted further development away from Fc-OPG as a RANK inhibitor, to Denosumab, a human monoclonal antibody against RANKL.^44, 45^

In addition to the 8q24.12 locus we identified a promising signal at 19q13.43 proximal to genes *ZNF444* and *GALP*. *GALP* encodes a member of the galanin family of neuropeptides that are active within the central nervous system.^46^ *ZNF444*, a zinc finger protein activates a scavenger receptor gene, which participates in the degradation of acetylated low-density lipoprotein.^47^ Intriguingly, acetylated low-density lipoprotein promotes osteoblastic differentiation, making *ZNF444* a credible candidate gene for the basis of the 19q13.43 MBD association.^48^

In making these conclusions, we have made use of various differing methods for MDB, which have different sensitivity for its detecting, the differences are between, not within, cohorts and hence systematic basis is unlikely to have impacted on our findings. In addition, the association was seen in each of the four patient cohorts and was not confined to a specific MM subtype. It is important to note, that the frequency of no MBD is equivalent in each of the four case series.

Bone loss in MM is unlikely to be exclusively attributable to RANKL/RANK/OPG providing a rationale for conducting further GWAS-based analyses to identify additional MBD risk variants. It is, however, noteworthy that SNPs, other than rs4407910, which are strongly associated with BMD, were not found by us to be associated with MBD. As our power to detect an allele with an odds ratio >1.5 was high (>90% power if the minor allele frequency>0.2), it implies that few such alleles are likely to exist. However our power to detect an association for an odds ratio ~1.2 is poor (that is, <20%), and such variants could readily exist. It is, therefore, apparent that larger studies will be required to identify additional risk loci for MBD. Although the impact of any individual genetic variant discovered by GWAS influencing MBD may be relatively modest, their identification serves to highlight genes and pathways relevant to developing novel intervention strategies. The genotyping of patients from ongoing and future clinical trials is likely to be especially informative in establishing the relationship between markers of MBD and specific therapies.

In summary, our observations provide the first evidence that germline variation influences a MM patient's risk of developing MBD and importantly impacts on this throughout disease progression. These results also provide further support for the importance of the RANK/RANKL/OPG pathway in the development of MBD. As no other established BMD locus showed an association with MBD, our results suggest the interaction between myeloma cells and bone remodeling is primarily dictated by this pathway. These findings will contribute to the development of future strategies for prevention of MBD by defining those MM patients at high risk of MBD and who may maximally benefit from therapeutic intervention.

## Figures and Tables

**Table 1:** Clinical characteristics and demographics of patients

	*My9*	*My11*	*HdB*	*ArK*
Number of cases	1205	768	1182	619
Median age at MM diagnosis	64	66	57	59

*Gender*				
Male	718 (59.6%)	446 (58.1%)	699 (59.1%)	389 (62.8%)
Female	487 (40.4%)	322 (41.9%)	483 (40.9%)	230 (37.2%)

*ISS*				
I	236 (20.8%)	181 (24.9%)	208 (44.2%)	293 (47.3%)
II	457 (40.3%)	290 (39.8%)	160 (34.0%)	182 (29.4%)
III	440 (38.8%)	257 (35.3%)	103 (21.9%)	144 (23.3%)
NA	72	40	711	0

*WHO performance stage*				
0	299 (25.0%)	265 (35.1%)	181 (48.1%)	NA
1	549 (45.9%)	319 (42.3%)	161 (42.8%)	NA
2	224 (18.7%)	130 (17.2%)	28 (7.4%)	NA
⩾3	125 (10.4%)	40 (5.3%)	6 (1.6%)	NA
NA	8	14	806	NA

*Bone disease*				
Yes	875 (72.6%)	554 (72.1%)	912 (77.2%)	457 (73.8%)
No	330 (27.4%)	214 (27.9%)	270 (22.8%)	162 (26.2%)

*Vertebral fractures*				
Yes	372 (41.3%)	222 (41.6%)	NA	NA
No	528 (58.7%)	312 (58.4%)	NA	NA
NA	305	234	NA	NA

*Heavy chain paraprotein*				
IgG	629 (62.2%)	403 (56.1%)	333 (58.7%)	353 (57.1%)
IgA	224 (22.1%)	195 (27.2%)	128 (22.6%)	140 (22.7%)
IgD	22 (2.2%)	13 (1.8%)	4 (0.7%)	4 (0.6%)
LCO	130 (12.8%)	103 (14.3%)	94 (16.6%)	111 (18.1%)
No paraprotein	7 (0.7%)	4 (0.6%)	8 (1.4%)	10 (1.6%)
NA	193	50	615	1

*Light chain paraprotein*				
Lambda	352 (34.7%)	239 (33.2%)	187 (33.1%)	238 (39.0%)
Kappa	662 (65.3%)	477 (66.3%)	378 (66.9%)	367 (60.0%)
No light chain	0	3 (0.4%)	0	6 (1.0%)
NA	191	49	617	8

*Hyperdiploidy*				
Yes	367 (55.6%)	222 (48.9%)	536 (55.9%)	NA
No	293 (44.4%)	232 (51.1%)	423 (44.1%)	NA
NA	545	314	223	NA

Abbreviations: ISS, international staging system; LCO, light chain only; MM, multiple myeloma; NA, not available.
