# Supplementary material for: Genetic factors influencing the risk of multiple myeloma bone disease
Source: Leukemia. 2016 Jan 12;30(4):883–8. doi: 10.1038/leu.2015.342 (PMC4832071; doi:10.1038/leu.2015.342)
Supplement: Supplementary Information [file leu2015342x1.doc]

**Genetic factors influencing the risk of multiple myeloma bone disease**

David C Johnson^1*^, Niels Weinhold^2,3*^, Jonathan Mitchell^4*^, Bowang Chen^5^, Owen W Stephens^2^, Asta Försti^5,6^, Jolanta Nickel^3^, Martin Kaiser^1^, Walter A Gregory^7^, David Cairns^7^, Graham H Jackson^8^, Per Hoffmann^9,10^, Markus M Noethen^9,11^, Jens Hillengass^3^, Uta Bertsch^3^, Bart Barlogie^2^, Faith E Davis^2^, Kari Hemminki^5,6**^, Hartmut Goldschmidt^3,12**^, Richard S Houlston^1,4**^, Gareth J Morgan^2**^

1. Division of Molecular Pathology, The Institute of Cancer Research, London, UK.
2. Myeloma Institute, University of Arkansas for Medical Sciences, Little Rock, AR, USA.
3. Department of Internal Medicine V, University of Heidelberg, Heidelberg, Germany.
4. Division of Genetics and Epidemiology, The Institute of Cancer Research, London, UK.
5. German Cancer Research Center, Heidelberg, Germany.
6. Center for Primary Health Care Research, Lund University, Malmö, Sweden.
7. Leeds Institute of Molecular Medicine, Section of Clinical Trials Research, University of Leeds, Leeds, UK.
8. Department of Haematology, Newcastle University, Newcastle-Upon-Tyne, NE1 7RU, UK.
9. Institute of Human Genetics, University of Bonn, Germany.
10. Division of Medical Genetics, Department of Biomedicine, University of Basel, Switzerland.
11. Department of Genomics, Life & Brain Center, University of Bonn, Germany.
12. National Center of Tumor Diseases, Heidelberg, Germany.

**SUPPLEMENTARY TABLES AND FIGURES**

**Supplementary Table 1: Relationship between SNP genotype and MBD (*P*_combined_<10^-5^)**

**Supplementary Table 2: Functional annotation with Haploreg and RegulomeDB of at 8q24.12 locus.** MBD associated SNPs in Bold.

**Supplementary Table 3: Summary expression quantitative trait loci of MBD risk regions.**

**Supplementary Table 4: MBD associations at reported GWAS BMD (*P*<10^-8^) loci.**  *Indicates where a proxy SNP has been used with r^2^=1 to associated variant. ** Genes proximal to other association regions. NA=not in data, with no suitable proxy SNP available. MBD associated SNPs in Bold.

**Supplementary Table 5: MBD associations at reported GWAS MM risk (*P*<10^-8^) loci.**

**Supplementary Figure 1: GWAS data quality control.** Details are provided of samples and SNPs used in UK-My9-GWAS, UK-My11-GWAS, German-HdB-GWAS and US-ArK-GWAS.

**Supplementary Figure 2: Identification of samples of non-European origin.** The first two principal components of the analysis were plotted for (a) UK-My9-GWAS and UK-My11-GWAS (b) German-GWA (c) US-ArK-GWAS. HapMap CEU individuals are plotted in blue; CHB individuals are plotted in magenta; JPT individuals are plotted in turquoise; YRI individuals are plotted in yellow; GWAS cases are plotted in red and controls are plotted in green.

**Supplementary Figure 3: Quantile-Quantile (Q-Q) plot for individual cohort analyses.** The y-axis corresponds to the observed -log_10_ *P*-value, and the x-axis the expected -log_10_ *P*-value. The red line represents the expected distribution under the null hypothesis of no association. All statistical tests were two-sided.

**Supplementary Figure 4: Quantile-Quantile (Q-Q) plot for the combined analyses.** The y-axis corresponds to the observed -log_10_ *P*-value, and the x-axis the expected -log_10_ *P*-value. The red line represents the expected distribution under the null hypothesis of no association. All statistical tests were two-sided.

**Supplementary Figure 5: Forest plots of the ORs for the association between rs4407910 and MBD in Male and Female patients only.** Horizontal lines represent 95% confidence intervals. Each box represents the OR point estimate and its area is proportional to the weight of the study. The diamond (and unbroken line) denotes the overall summary estimate, with CIs given by its width. The unbroken vertical line is at the null value (OR = 1.0)

**Supplementary Figure 6: Forest plots of the ORs for the association between rs4407910 and MBD in Hyperdiploids and Non-Hyperdiploids patients only.** Horizontal lines represent 95% confidence intervals. Each box represents the OR point estimate and its area is proportional to the weight of the study. The diamond (and unbroken line) denotes the overall summary estimate, with CIs given by its width. The unbroken vertical line is at the null value (OR = 1.0)

**Supplementary figure 7: Encode annotation of the 8q24.12 locus.** Functional annotation by ENCODE including DNAse1 Hypersensitivity analysis and Regulatory protein bound regions and histone modifications indicated by ChIP-seq of H3K4m1, H3K4m2, H3K4m3, H3K27ac, H3K27m3, H3K36m3, H3K79m2, H4K20m1, P300, CTCF in osteoblast cells (in greyscale light (lowest) to dark (highest))

**Supplementary Figure 8: Regional plot of association and recombination rates for the 19q13.43 locus (rs74676832).** Association results of both genotyped (triangles) and imputed (circles) SNPs and recombination rates. −log_10_*P* values (*y* axes) of the SNPs are shown according to their chromosomal positions (*x* axes). rs74676832 shown as a large diamond. The color intensity of each symbol reflects the extent of LD with rs74676832 white (*r*^2^ = 0) through to dark red (*r*^2^ = 1.0). Genetic recombination rates, estimated using HapMap samples from Utah residents of western and northern European ancestry (CEU), are shown with a light blue line. Physical positions are based on NCBI build 37 of the human genome. Also shown are the relative positions of genes and transcripts mapping to the region of association

**Supplementary Table 1: Relationship between SNP genotype and MBD (*P*_combined_<10^-5^)**

**Supplementary Table 2: Functional annotation with Haploreg and RegulomeDB of the 8q24.12 locus.**

**a) Haploreg**

**b) RegulomeDB**


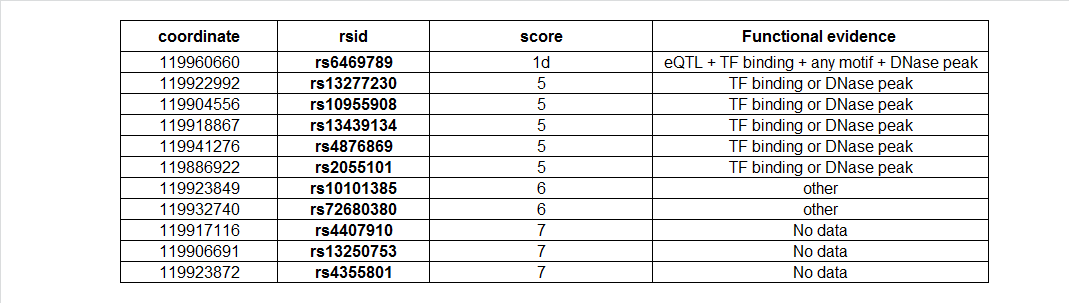


**Supplementary Table 3: Summary expression quantitative trait loci of MBD risk at 8q24.12 locus.**

^1^Richards, J.B . et al - Lancet, 2008; ^2^Ge, B . et al - Nature Genetics, 2009; ^3^Grundberg, E. et al - Nature Genetics, 2012; ^4^Liang, L. et al - Genome Research, 2013; ^5^Grundberg , E. et al - Genome Research, 2009; ^6^Hsu, Y. et al - PLoS Genetics, 2010; ^7^Grundberg, E. et al - PLoS Genetics, 2011.

**Supplementary Table 4: MBD associations at reported GWAS BMD (*P*<10^-8^)** **loci**


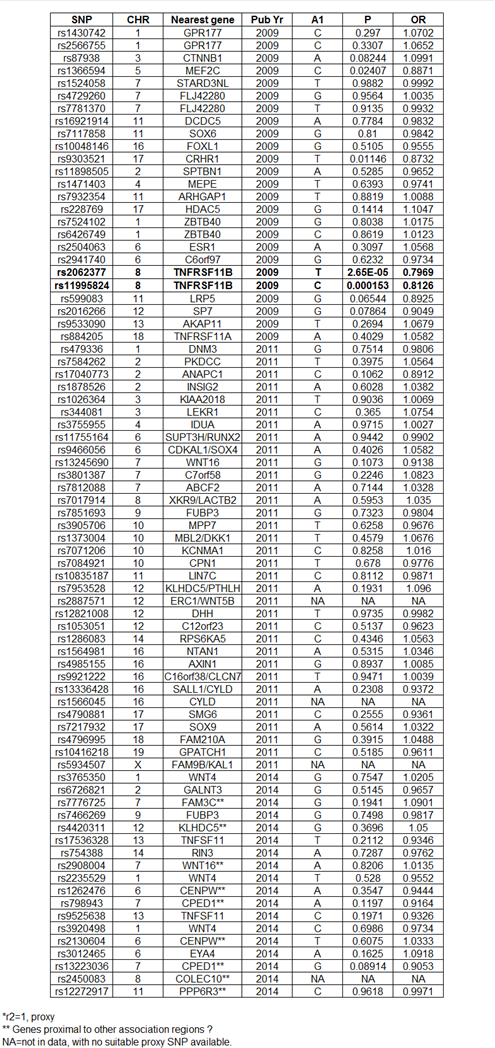


**Supplementary Table 5: MBD associations at reported GWAS MM Risk (*P*<10^-8^)** **loci**

^a^Odds ratio, ^b^*p*-value for Cochrane's Q statistic, ^c^Heterogeneity index. ^8^Broderick, P. et al - Nature genetics, 2011; ^9^Erickson, S.W .et al - Blood, 2014; ^10^Chubb, D. et al - Nature genetics, 2013; ^11^Swaminathan, B. et al - Nature Communications, 2015.

**Supplementary Figure 1: GWAS data quality control.**

**Supplementary Figure 2: Identification of samples of non-European origin.**

**Supplementary Figure 3: Quantile-Quantile (Q-Q) plot for individual cohort analyses. a) My9 b) My11 c) HdB d) ArK.**

a) My9 b) My11


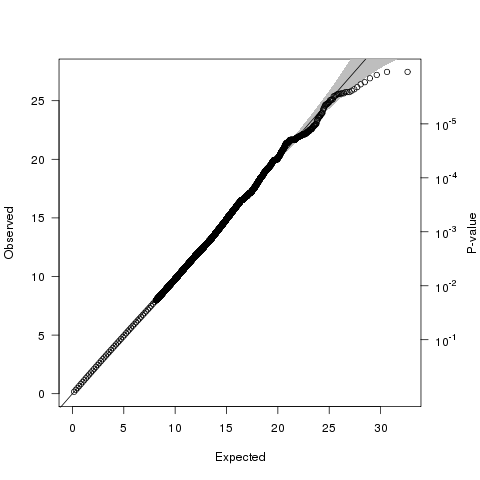

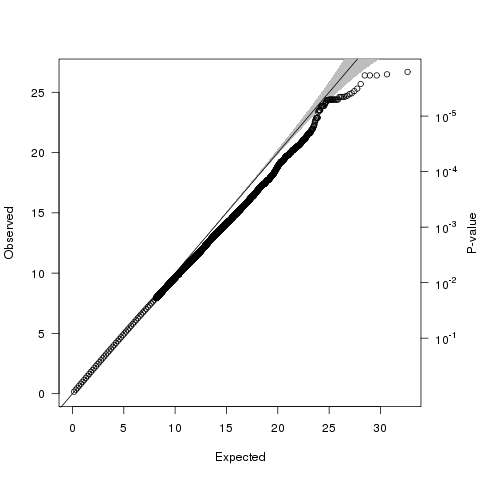


c) HdB d) ArK


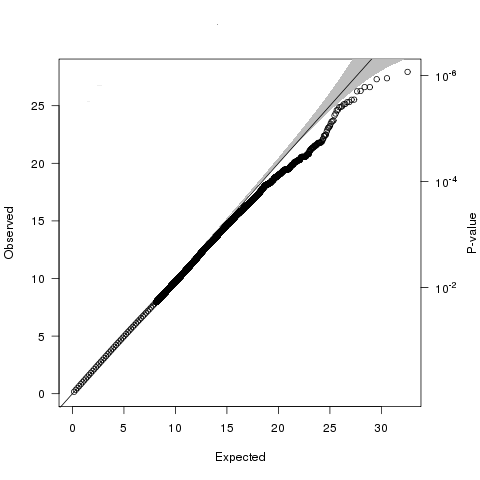

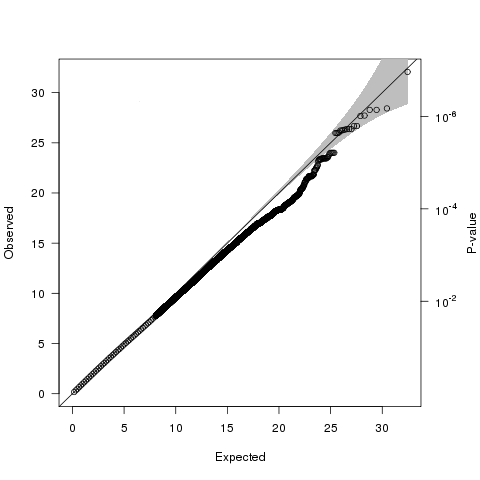


**Supplementary Figure 4: Quantile-Quantile (Q-Q) plot for the combined analyses.**

**
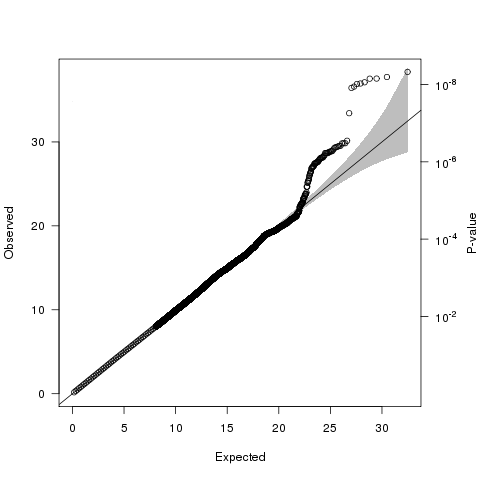
**

**Supplementary Figure 5: Forest plots of the ORs for the association between rs4407910 and MBD in Male and Female patients only**

i) Females

**
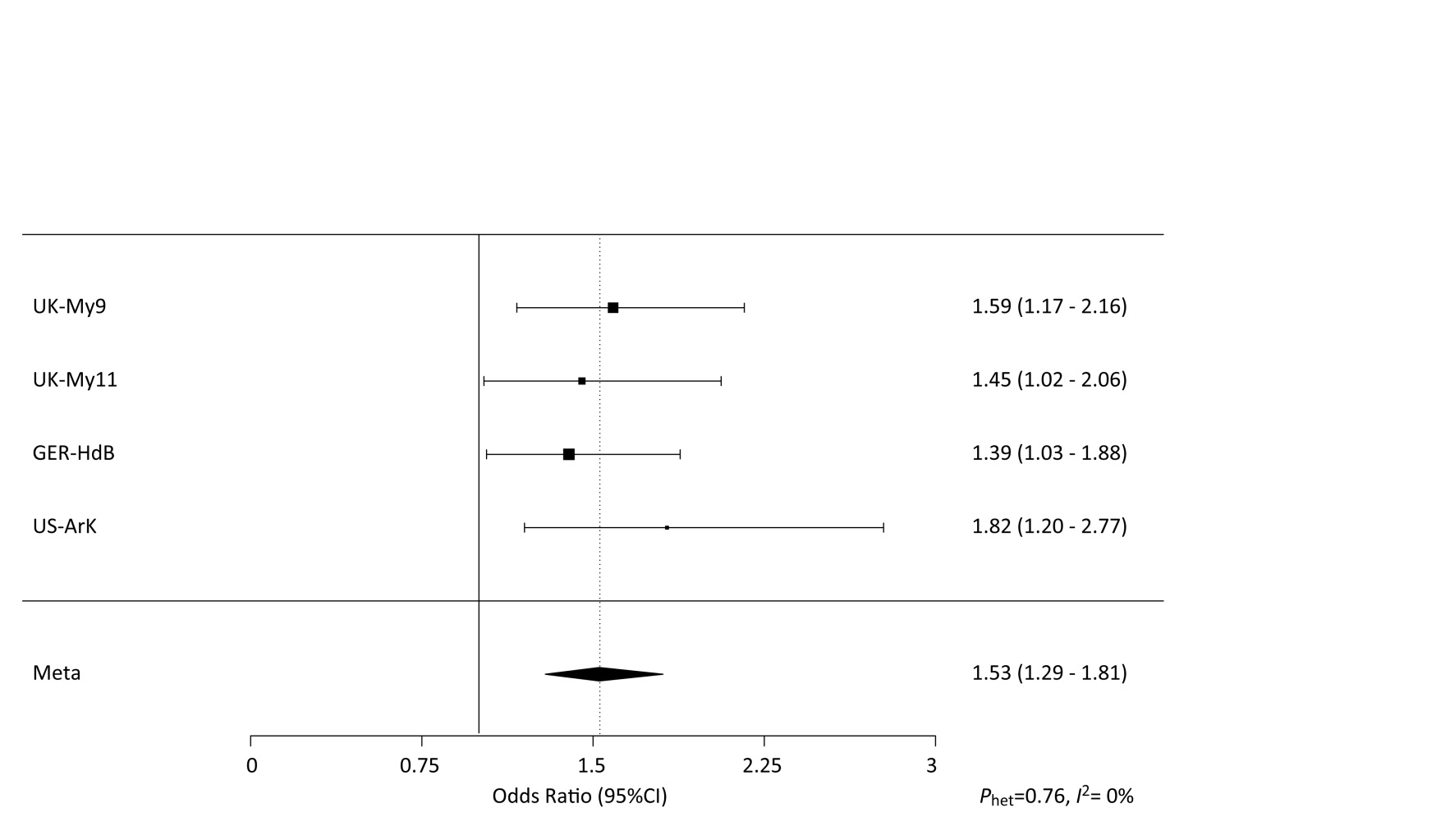
**

ii) Males

**
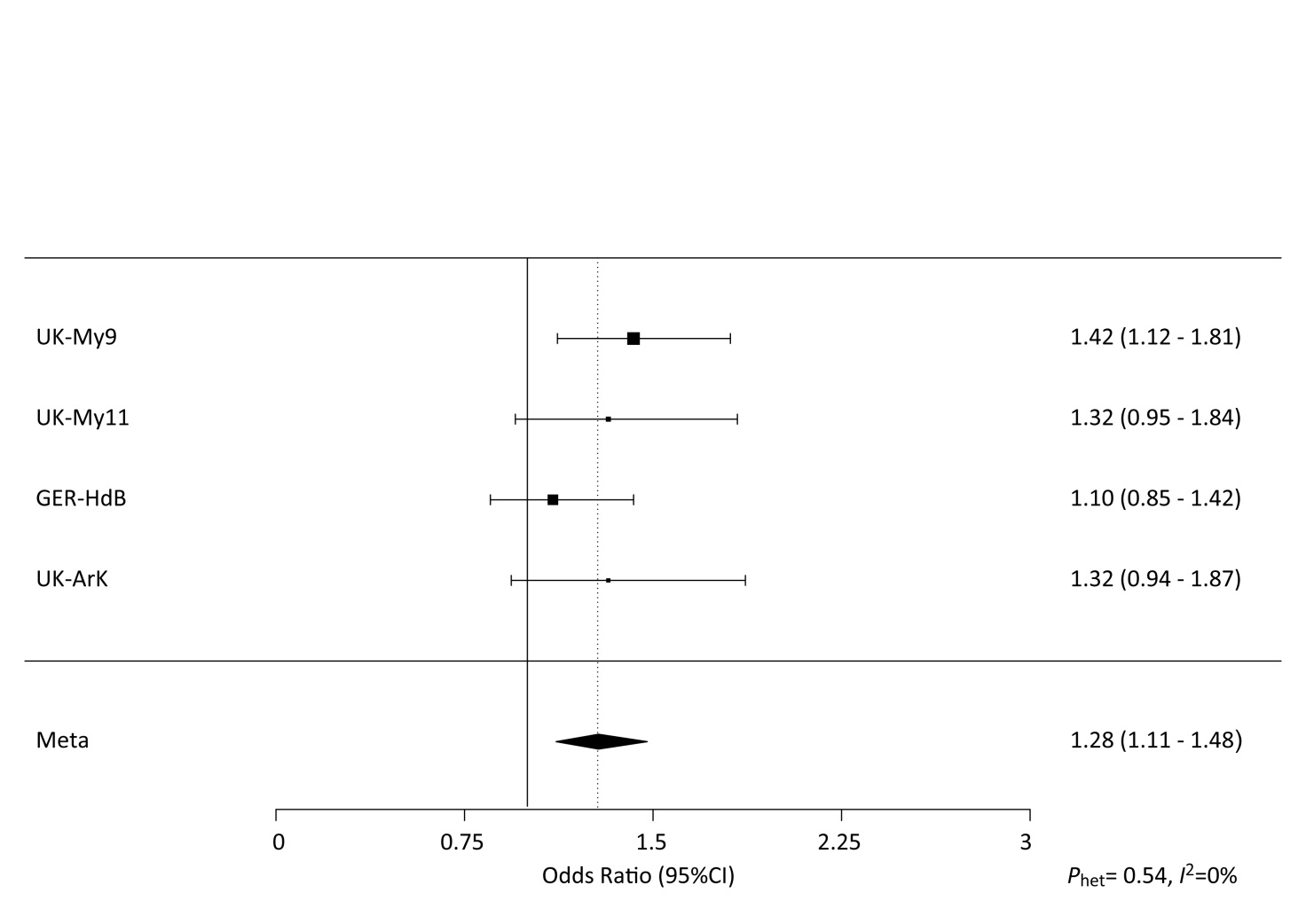
**

**Supplementary Figure 6: Forest plots of the ORs for the association between a) rs4407910 and MBD in Hyperdiploids and Non-Hyperdiploids patients only.**

i) Hyperdiploids

**
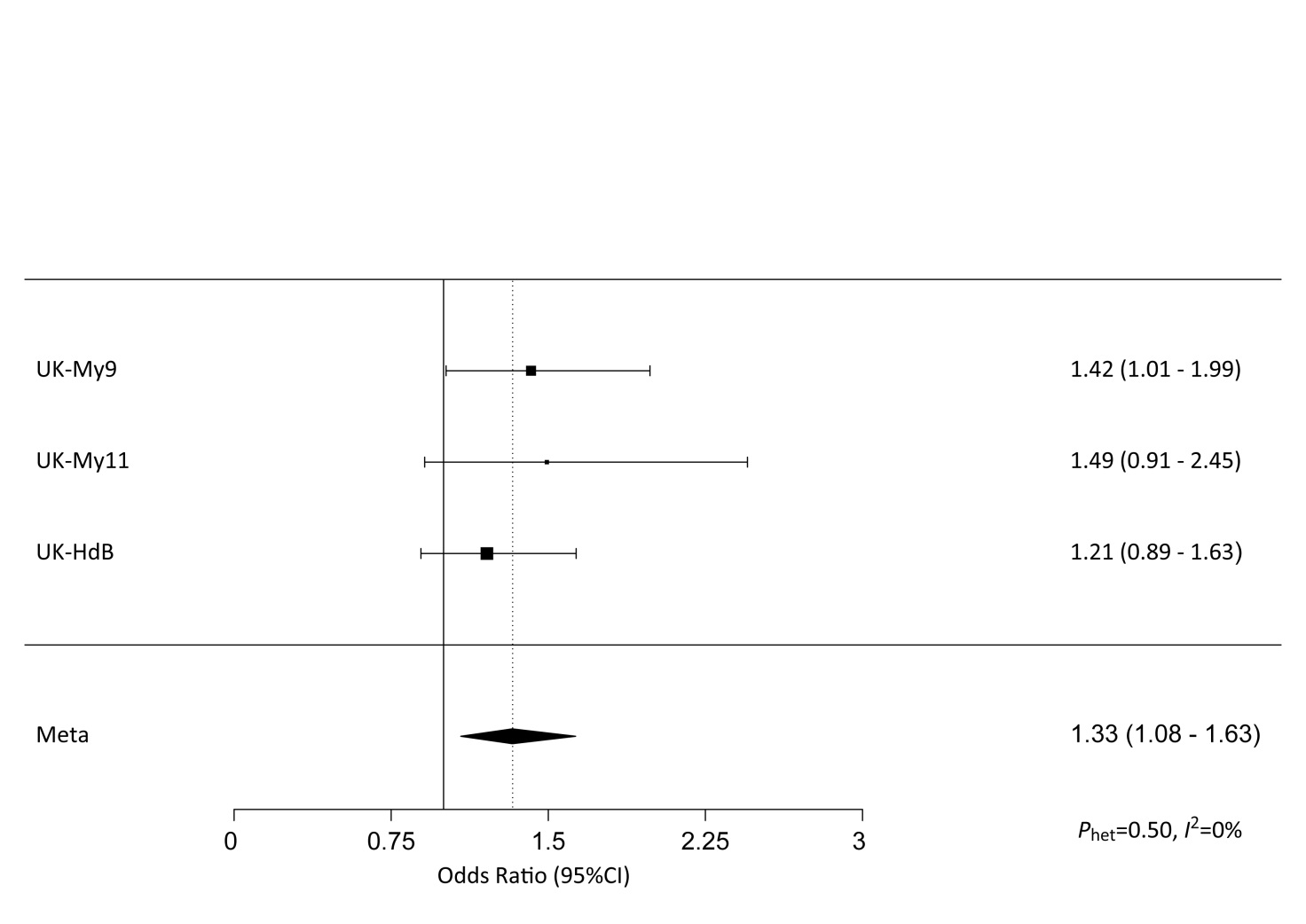
**

ii) Non-Hyperdiploids

**
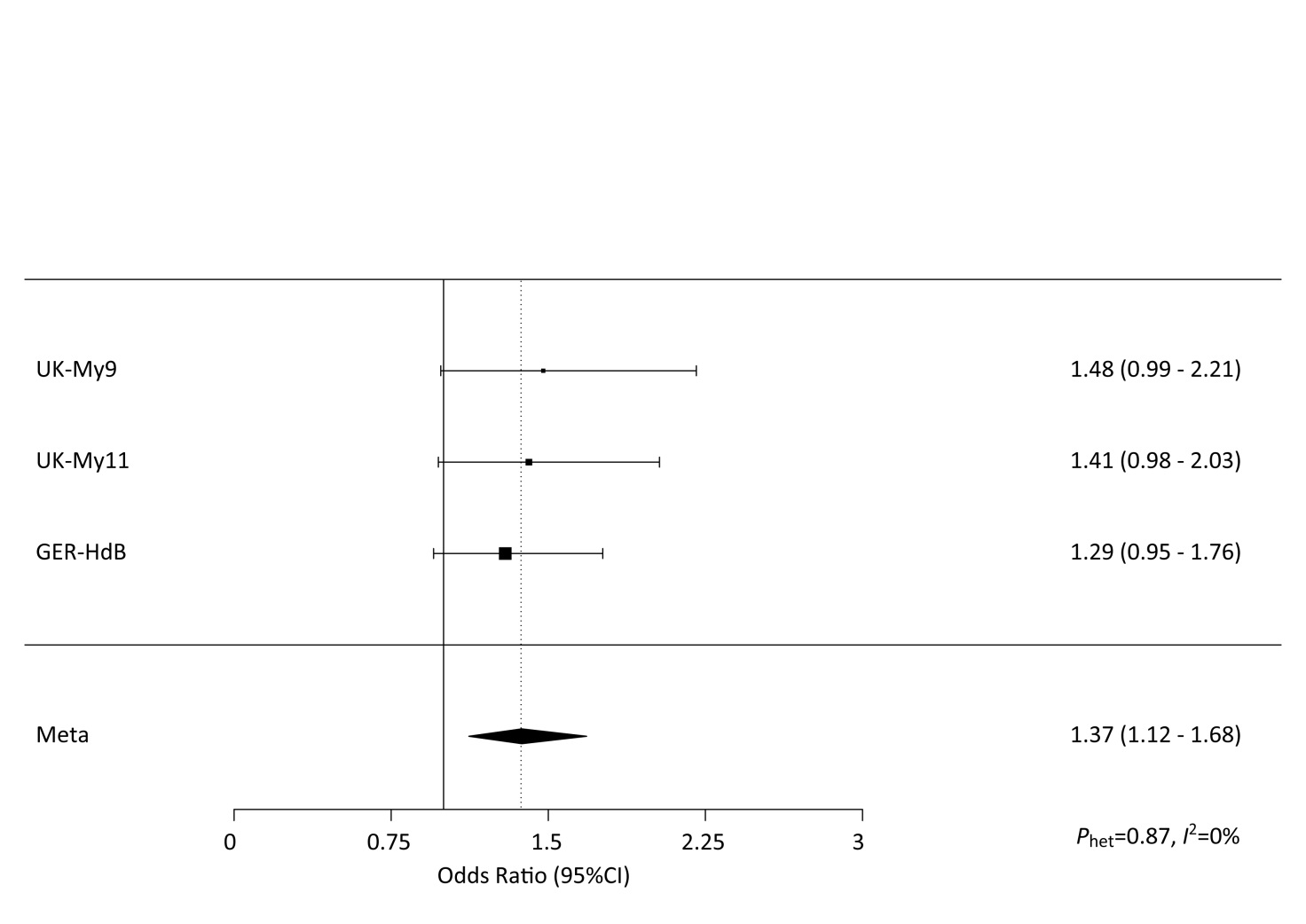
**

**Supplementary Figure 7: Encode annotation of the 8q24.12 locus**


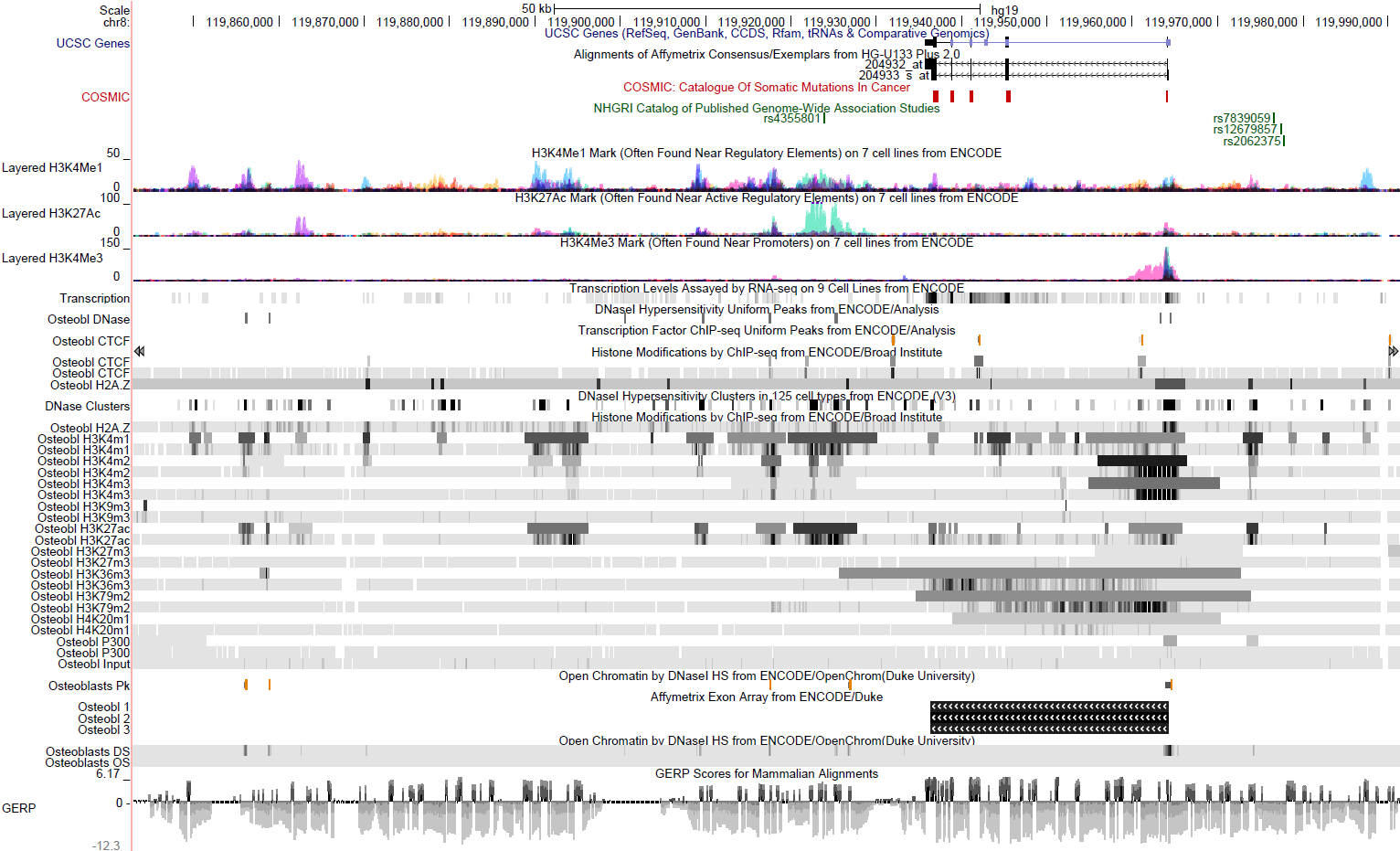


**Supplementary Figure 8: Regional plot of association and recombination rates for the 19q13.43 locus (rs74676832).**


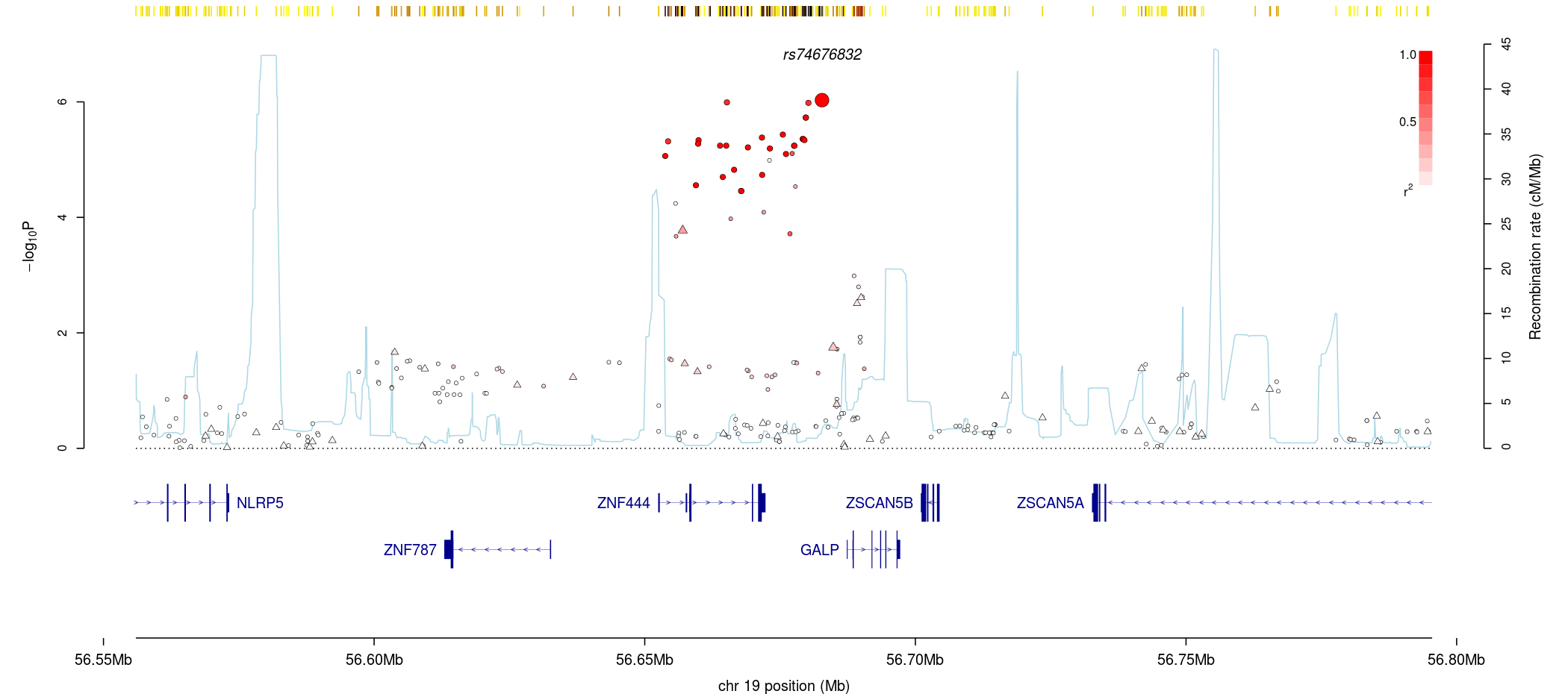


**Supplementary References**

1. Richards, J.B. *et al.* Bone mineral density, osteoporosis, and osteoporotic fractures: a genome-wide association study. *Lancet* **371**, 1505-12 (2008).

2. Ge, B. *et al.* Global patterns of cis variation in human cells revealed by high-density allelic expression analysis. *Nat Genet* **41**, 1216-22 (2009).

3. Grundberg, E. *et al.* Mapping cis- and trans-regulatory effects across multiple tissues in twins. *Nat Genet* **44**, 1084-9 (2012).

4. Liang, L. *et al.* A cross-platform analysis of 14,177 expression quantitative trait loci derived from lymphoblastoid cell lines. *Genome Res* **23**, 716-26 (2013).

5. Grundberg, E. *et al.* Population genomics in a disease targeted primary cell model. *Genome Res* **19**, 1942-52 (2009).

6. Hsu, Y.H. *et al.* An integration of genome-wide association study and gene expression profiling to prioritize the discovery of novel susceptibility Loci for osteoporosis-related traits. *PLoS Genet* **6**, e1000977 (2010).

7. Grundberg, E. *et al.* Global analysis of the impact of environmental perturbation on cis-regulation of gene expression. *PLoS Genet* **7**, e1001279 (2011).

8. Broderick, P. *et al.* Common variation at 3p22.1 and 7p15.3 influences multiple myeloma risk. *Nat Genet* **44**, 58-61 (2012).

9. Erickson, S.W. *et al.* Genome-wide scan identifies variant in 2q12.3 associated with risk for multiple myeloma. *Blood* **124**, 2001-3 (2014).

10. Chubb, D. *et al.* Common variation at 3q26.2, 6p21.33, 17p11.2 and 22q13.1 influences multiple myeloma risk. *Nat Genet* **45**, 1221-5 (2013).

11. Swaminathan, B. *et al.* Variants in ELL2 influencing immunoglobulin levels associate with multiple myeloma. *Nat Commun* **6**, 7213 (2015).
